# Push out bond strength of hydraulic cements used at different thicknesses

**DOI:** 10.1186/s12903-023-02758-w

**Published:** 2023-02-07

**Authors:** C. Ruiz Durán, Dra L. Gancedo-Caravia, V. Vera González, C. González Losada

**Affiliations:** Department of Consevative and Prosthetic Dentistry, School of Dentistry, Complutense University, Pza de Ramón y Cajal s/n, Ciudad Universitaria, 28040 Madrid, Spain

**Keywords:** Hydraulic cement, Totalfill, Biodentine, MTA, Pushout bond strength, Weilbull analysis

## Abstract

**Background:**

The aim of this study was to compare the pushout bond strength (POBS) of three hydraulic cements, when used at thicknesses of 3 and 5 mm.

**Methods:**

78 root slices of 3 and 5 mm of thickness were obtained from human teeth. Cylindrical cavities of 1.4 mm of diameter were drilled and filled with Biodentine (BD), Totalfill Root Repair paste (TF) or ProRoot MTA White (PMTA). Pushout tests were performed 21 days later. The fracture pattern of each sample was also analyzed. POBS data were analyzed with Welch and Brown-Forsythe and Tamhane’s post hoc tests and a Weibull analysis was also performed.

**Results:**

In the 3 mm group, TF showed significantly lower bond strength than BD and PMTA. In the 5 mm group, BD showed significantly higher bond strength than TF. Both BD and TF showed higher bond strength when the thickness of the sample increased, while PMTA did not.

**Conclusions:**

TF and BD achieve higher pushout bond strength resistance when used at a thickness of 5 mm than at 3 mm, while the mean resistance of PMTA is less influenced by the thickness. At 5 mm of thickness, BD and PMTA exhibit similar resistance to displacement. However, the behavior of BD is more predictable than that of its predecessor. BD is a reliable hydraulic cement for clinical situations where thick cavities need to be filled and displacement resistance plays an important role. Clinicians need to consider choosing specific hydraulic cements according to the thickness of material to be used.

**Supplementary Information:**

The online version contains supplementary material available at 10.1186/s12903-023-02758-w.

## Background

Hydraulic cements are a group of materials that become hydrated as they contact water-based fluids [1]. Depending on the characteristics of the medium in which these cements are used, either calcium carbonate or apatite precipitates can be formed [2]. The term hydraulic is the most accepted way to refer to these materials, as it describes their chemistry and clinical behavior [1].

Mineral trioxide aggregate (MTA) was the first hydraulic cement used in endodontics. Since it was first described by Lee and Torabinejad in 1993 [3] and commercialized in 1999 under the name ProRoot MTA (Dentsply, Baillages, Switzerland), it has been the most widely used material in endodontics.

Despite its proven superiority when compared to previous materials, MTA has some shortcomings, such as complex handling, long setting time and it also may induce tooth discoloration. Some of these problems can be explained by the composition of MTA. As an example, bismuth oxide, which is meant to provide MTA with radiopacity, is responsible for the change of color in treated teeth, due to its dissociation when MTA is exposed to ultraviolet light, and results in the formation of reduced dark crystals that yield to discoloration [4] Bismuth oxide tends to dissolve in the acidic media of perirradicular lesions, resulting in a decreased biocompatibility of MTA, and it also contributes to alter the calcium hydroxide precipitation process that occurs during the hydration of the cement [5].

New materials have been developed to overcome the problems found using MTA. As a result of modifications in the composition of more classic cements and improvements achieved in the field of nanotechnology a new generation of cements have been released to the market. Among these new materials are Totalfill (FKG Dentaire, La Chaux-de-Fonds, Switzerland. Commercialized as EndoSequence Root Repair Material in USA) (TF) and Biodentine (Septodont, Saint-Maur-des-Fossés, France) (BD). As one of its improvements, TF uses zirconium oxide and tantalum as radiopacifiers, which are not associated with tooth discoloration [6]. BD also contains zirconium oxide instead of bismuth oxide, managing to avoid the discoloration problem [7, 8]. There are also improvements when it comes to working and setting time. TF allows a working time of thirty minutes, compared with the five minutes of MTA, and the setting reaction of TF is completed within 4 h [9]. The setting time of BD has been drastically reduced to twelve minutes, thanks to the incorporation of calcium chloride as an accelerator [10, 11]. TF offers improved handling properties, as it comes in ready-to-use syringes with application cannulas [9, 12], and BD has also been reported to be an easy-to handle-product. [13, 14]

The main goal of endodontic treatment in necrotic teeth with open apices is to induce apical closure, either by stimulating the apposition of calcified tissues with calcium hydroxide or by using materials to create an artificial plug at the apical end of the root canal [15]. The use of MTA for building apical plugs helps diminish the weakening of the tooth associated with the use of calcium hydroxide for extended periods [16]. It also requires a shorter treatment time, which leads to a higher level of patient compliance and therefore to higher success rates [17, 18]. Another application of hydraulic cements is to provide a coronal barrier in regenerative endodontic procedures. MTA has been widely used for this purpose [18, 19] and the success of the treatment has been attributed to the optimal sealing ability of the cement, as well as the good biocompatibility and conductive and inductive properties [18, 20]. MTA is also currently considered the material of choice for retrograde sealing after periapical surgery as it achieves more favorable results and better clinical outcomes when compared to other traditionally used materials, such as Super EBA and amalgam [18, 21]. Based on the results of studies focused on physical properties, biocompatibility and clinical performance, both TF and BD seem to be favorable alternatives to MTA when performing periapical surgery and endodontic treatment of immature teeth [12, 14, 22–27]. However, there are still certain aspects in their mechanical performance that have not been thoroughly contrasted yet.

To ensure optimal sealing when using hydraulic cements in the clinical situations previously described, a thickness of 3 to 5 mm has been advocated by manufacturers as well as by several authors [9, 13, 28–30]. However, there is not enough quality information to predict and compare the performance of the different materials at these suggested thicknesses, and therefore it is not possible to establish if this variable plays a determinant role in treatment outcomes.

For all the mentioned clinical applications, a repairing material needs to provide not only an excellent seal, but also sufficient bonding strength to resist dislodging forces caused by restorative processes and by chewing movements. Push-out tests are used in dentistry to evaluate the bonding strength of a material to the tooth. There are several studies that use push-out bond strength (POBS) as a method to evaluate the performance of hydraulic cements for several clinical applications and scenarios [26, 31–39]. Most studies use mean POBS results to infer the expected outcome with materials in the different conditions tested. However, it is well known that mechanical behavior of materials used in dentistry is not predictable, usually due to problems that occur during the handling or preparation process [40]. In addition, these material defects are heterogeneous and can be caused by presence of pores, microcracks or defects of different sizes [41]. Thus, further analysis of hydraulic cements and their displacement resistance may provide additional information to evaluate and compare their suitability as repairing materials. To the authors´ knowledge, such analysis has not been conducted so far with hydraulic cements.

The aim of this study is to compare the push-out bond strength (POBS) of three hydraulic cements (BD, TF and PMTA), when used at thicknesses of 3 and 5 mm.

The null hypothesis states the three analyzed hydraulic cements have similar POBS values regardless of the thickness employed.

## Materials and methods

This study was conducted with the approval of the Ethics Committee of The Clinical Hospital of San Carlos in Madrid, Spain (19/052-E-Tesis). Informed consent to manipulate extracted teeth was obtained from all subjects and/or their legal representative.

A total of 156 mature, single-rooted extracted teeth were selected with the following inclusion criteria: they did not have cavities, resorption defects, fractures or root fissures and they had not undergone any previous endodontic treatment. The number of samples was established according to a previous pilot study designed to calculate the sample size. In this study we considered a significance *p* = 0.05, statistical power of 80% and a typical deviation of 0.5 MPa.

The specimens were randomly divided in two groups (A and B) and preserved in a saline solution for a maximum time of 6 months.

Teeth were sliced with a diamond band cutting machine (300 CL. Exakt, Hamburg, Germany). The apical three millimeters of the roots were removed and discarded. Subsequently, 3 mm sections (group A) and 5 mm sections (group B) were cut. Section thickness (± 0.05 mm) was verified with a digital vernier caliper (500–181-30. Mituitoyo, Berlin, Germany). Cavities with a diameter of 1.4 mm were drilled in the samples with a cylindrical diamond bur.

A series of specimen frames, sample holders and fixation devices were used to prepare the samples for the push-out test. These devices had been designed and tested in previous studies [37, 38, 42].

**Fig. 1:**
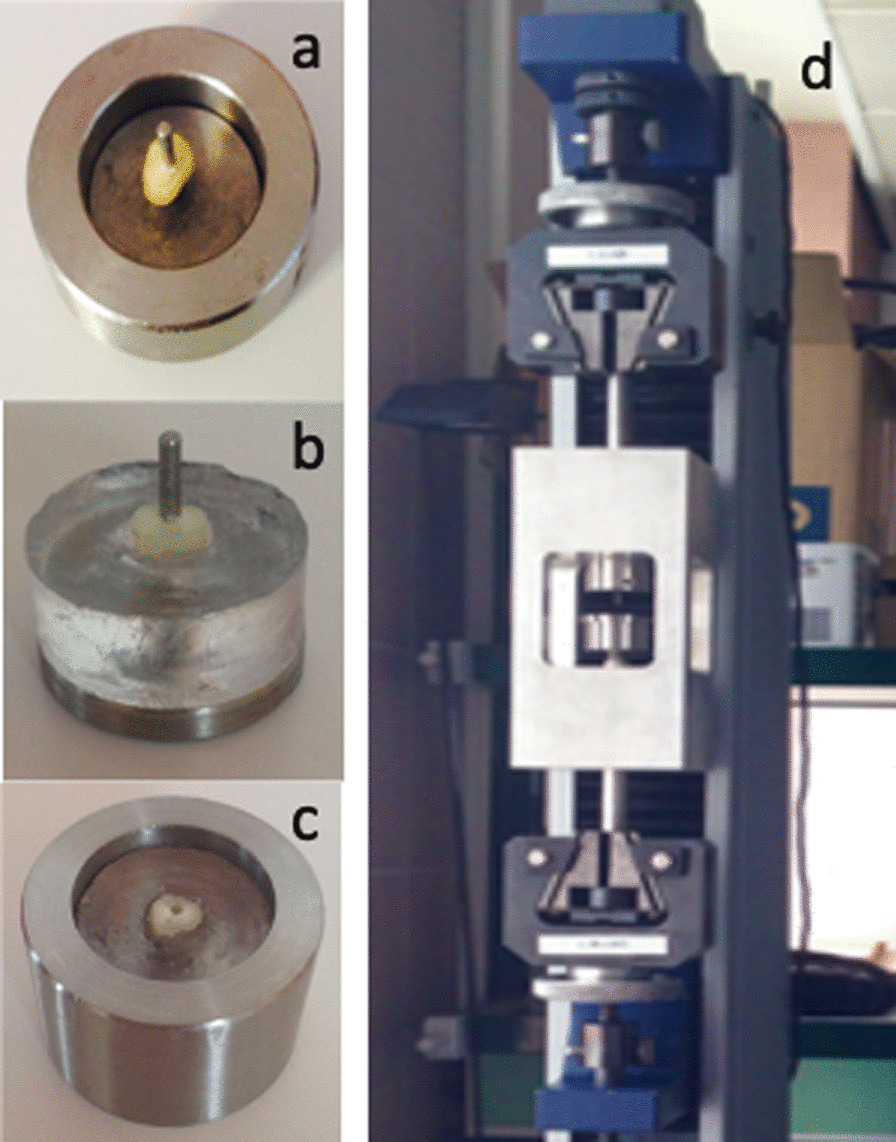
Specimen preparation and push-out test devices. **a** Cylindrical steel frame. Inside, a sample with the dentin aligned with the major axis. **b** Sample with the root slice located on the upper side and embedded in resin. **c** Sample holder with one specimen inside. **d** Sample holder and punch aligned inside the Universal Testing Machine

Root slices were placed in stainless steel frames and were later embedded in resin (Fig. 1a, b). The resulting specimens were resin cylinders that held the samples on the upper surface and kept the central cavity aligned with the major axis of the cylinder (Fig. 1c).

In group A (n = 78), the 3 mm-thick dentine fragments were randomly divided into three subgroups of 26 samples, according to the material used to fill the cavities (TF, BD or PMTA).

Similarly, group B (n = 78) comprised 5 mm samples that were randomly assigned to be filled with the same three materials as in group A (n = 26).

The cements were mixed following the instructions provided by manufacturers and then were used to fill the cavities. After that process, the specimens were stored for 21 days inside an incubator at 37ºC and 100% relative humidity to allow setting of the cements.

**Fig. 2:**
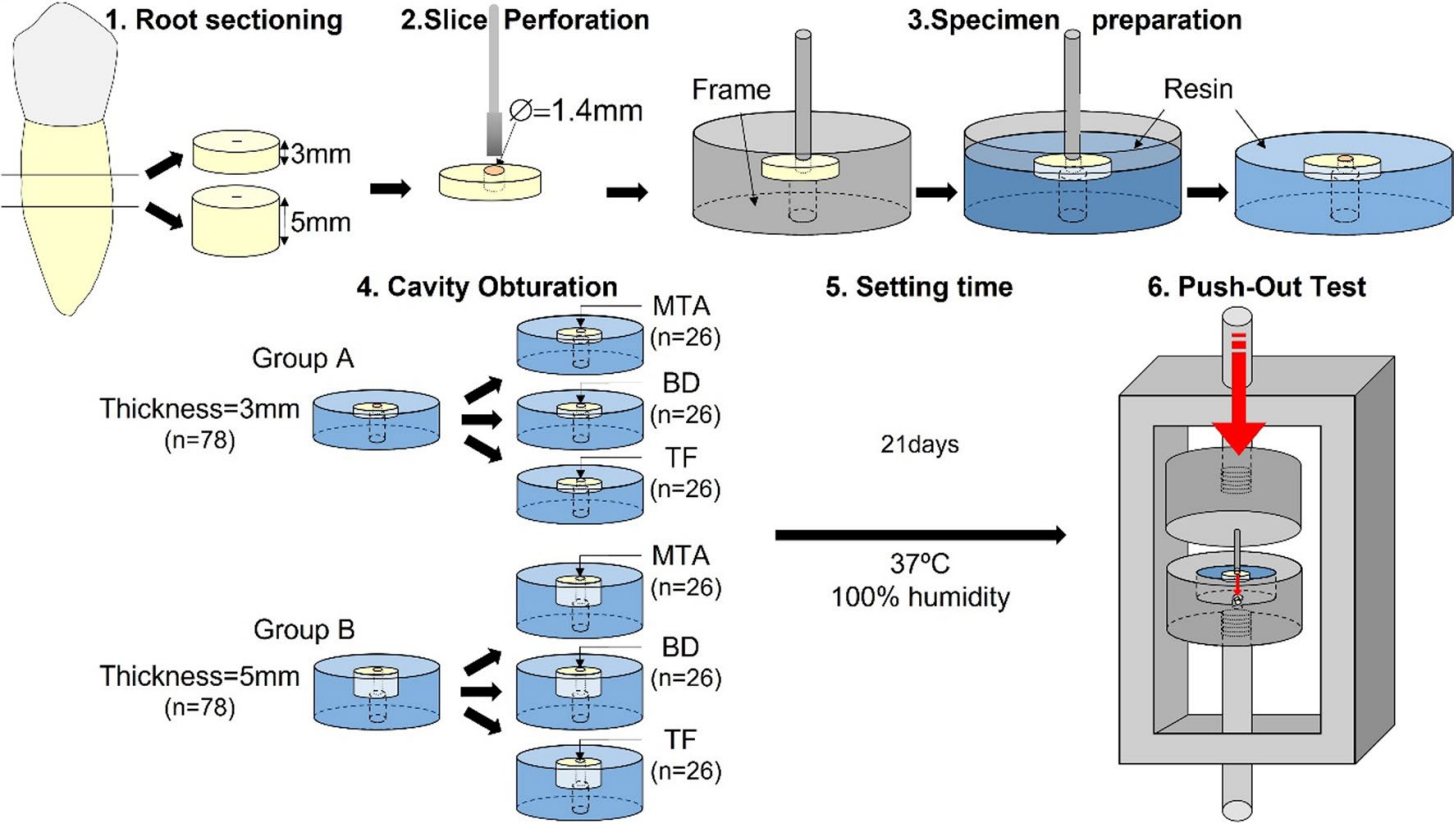
Schematic representation of the experimental protocol

After the setting time, the specimens were transferred to an aligning device that allowed the sample to be positioned in line with a 1, 2 mm diameter stainless-steel punch. The device with all the pieces in place was then attached to a Universal Testing Machine (Hounsfield h 5000 M, Metrotec, Lezo, Spain) that had previously calibrated by a third party. (Fig. 1d). Figure 2 summarizes the experimental protocol followed.

A progressively increasing force was exerted onto the punch until the cement-dentin bond was broken. Fractures developed in a trajectory parallel to the dentin-cement interface, allowing evaluation of the force necessary for dislocation. To obtain the push-out bond strength (POBS) values (MPa), the maximum force (F) of each sample (registered in Newtons) was divided by the contact area between the material and the cavity wall (S) applying the following conversion formula: Resistance (MPa) = F (N)/S (mm2) × 10–6 m2 × 10^–6^ Pa. And S was obtained with the following formula: S = 2 × r (mm) × π × h (mm), where r is the radius of the perforation, π is the constant 3.14, and h is the thickness of the slice (h = 3 mm in group A and h = 5 mm in group B). After performing the push-out test the specimens were observed under stereomicroscope at 40 × magnification and the adhesive pattern was categorized as: adhesive (in the dentin-material interface), cohesive (within the material) or mixed (combination of both adhesive and cohesive patterns).

Statistical significance was established at p = 0.05 Normality of data distribution was assessed with Shapiro–Wilk test. Afterwards, mean POBS of the different groups were compared with Welch and Brown-Forsythe tests, as well as Tamhane's post-hoc tests. A Student’s t-test was performed for each material to compare the mean POBS results obtained with the two thicknesses. Also, a Weibull analysis was performed to evaluate the predictability of the materials’ behavior at each thickness.

To evaluate the distribution of fracture patterns among the different groups, a Chi Square test was used.

## Results

**Fig. 3:**
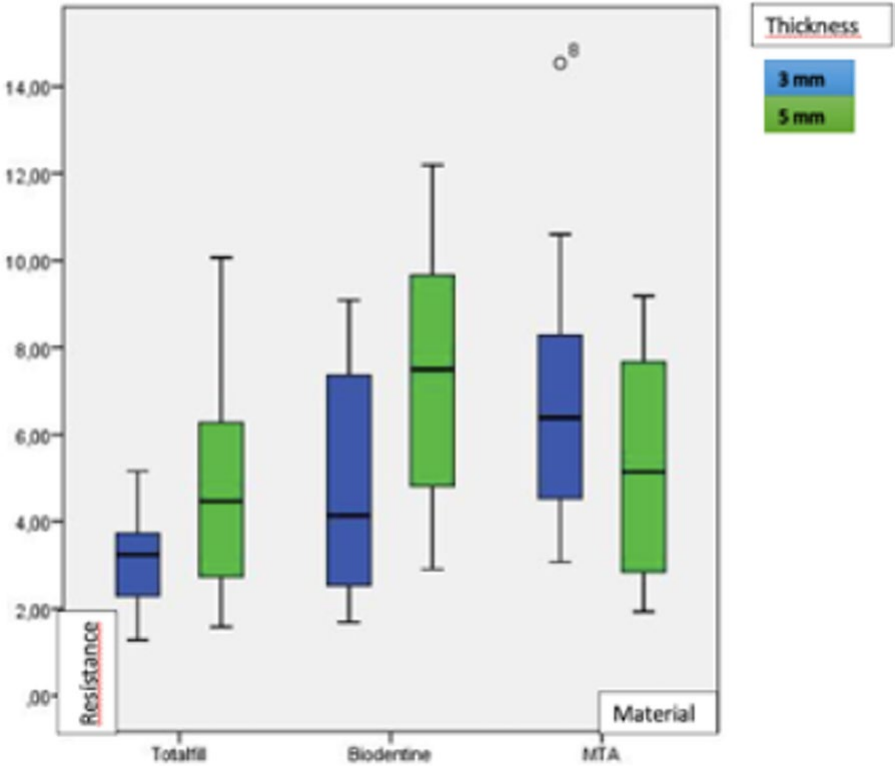
Box plot graphic showing the POBS of the six experimental groups Total Fill, Biodentine and ProRoot MTA at 3 and 5 mm

Mean POBS and standard deviation of BD, TF, and PMTA at 3 and 5 mm thicknesses are shown in Table 1. In the 3 mm group, Welch and Brown-Forsythe tests revealed significant differences among the three materials (*p* < 0.001) and according to Tamhane’s T2 post hoc test, TF showed significantly lower POBS than the other two materials (*p* < 0.001), while no statistically significant differences were found between BD and PMTA groups (*p* = 0.147). In the 5 mm group, a significant difference was also observed (*p* ≤ 0.003) and Tamhane’s T2 post hoc test revealed a significant difference between the group with the greatest (BD) and the group with the lowest mean values (TF) (*p* = 0.001). Figure 3 shows box plot graphics representing the POBS results of the six study groups. Student’s t-test determined that increasing the thickness significantly increased the POBS of TF (*p* = 0.044) and BD (*p* = 0.021). For PMTA there were no significant differences in the POBS achieved at 3 and 5 mm thickness (*p* = 0.075).

**Table 1:** Mean and standard deviation (SD) of POBS values of Total Fill, Biodentine and ProRoot MTA at 3 and 5 mm

Thickness	Totalfill		Biodentine		ProRoot MTA	
	POBS (MPa)		POBS (MPa)		POBS (MPa)	
	Mean	(SD)	Mean	(SD)	Mean	(SD)
3 mm	4.14819	(1.902544)^a^	6.65019	(2.203267)^b^	8.04996	(2.815383)^b^
5 mm	5.43785	(2.545617)^a,b^ *	8.07708	(2.113398)^a,b,c^ *	6.53865	(3.166277)^b,c^

^a,b^For the same thickness, different superscript small letters represent statistically significant differences in POBS values among cements

*For the same cement, statistically significant difference in POBS between thicknesses

Weibull analysis determined that in the 3 mm groups, there were no differences regarding the shape parameter (m) among the three cements, which represents the slope of the line of the accumulated Weibull probability graphic and is related to the predictability of the material. In samples of group B (5 mm) this parameter was greater for BD (4.37) than for TF (2.5) and PMTA (2.2).

**Fig. 4:**
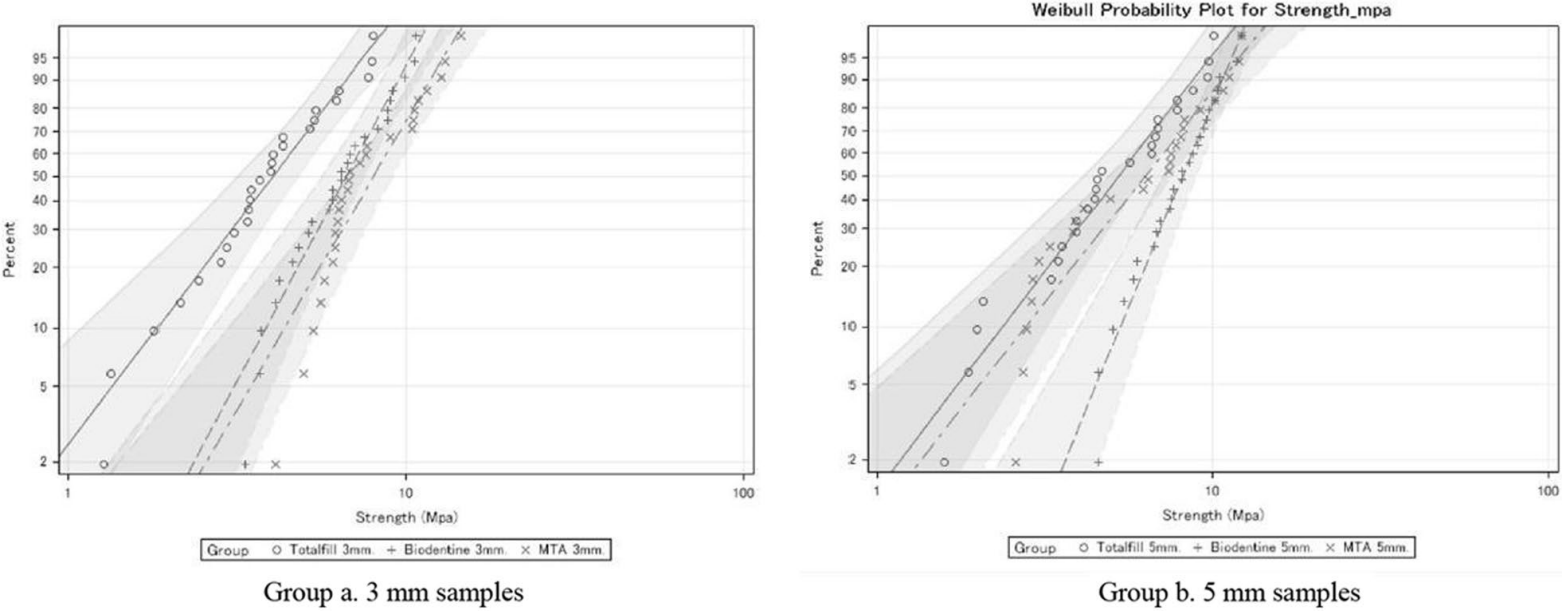
Weibull probability distribution per groups **a** 3 mm thickness. **b** 5 mm thickness

The scale parameter of the Weibull analysis (σ0), for both the 3 mm and 5 mm groups was lower for TF (4.7/6.1 MPa) than for BD (7.4/8.8 MPa) and PMTA (9/7.4 MPa), which means that two-thirds of the samples filled with TF needed a lower force to be detached than those filled with the other two cements, a fact that is consistent with the results obtained with parametric tests. The distributions of probability of failure in relation with the bond strength measured for the two thicknesses are shown in Fig. 4.

The total distribution of the different fracture patterns in all the specimens was as follows: 87.82% mixed, 8.9% adhesive and 3.28% cohesive. Chi-square test revealed no differences in the distribution of fracture patterns among groups.

## Discussion

One of the objectives of root canal treatment is to seal possible communications between the pulp space and the external surface of the tooth. If the mechanical resistance of the dentin-repairing material interface is low, the material will easily be displaced from the cavity and treatment will be more likely to fail. ProRoot MTA White is considered the gold standard when comparing hydraulic cements. The objective of this study was to compare this first material with two other recently developed cements in order to verify whether their new features have improved their mechanical behavior and they can stand as a real alternative to MTA in terms of bond strength.

The bond strength was tested after a storage time of 21 days to ensure complete setting t of the cements, based on previous studies that suggest a prolonged hydration process of these materials [33, 37, 42, 43].

Most clinicians perform apical or coronal barriers of random thicknesses between 3 and 5 mm, which are the recommended thicknesses for apexification, endodontic surgery or revitalization procedures. Several studies have evaluated the sealing ability of hydraulic cements at different thicknesses and have established that a 3 to 5 mm-thick plug can produce an acceptable seal [29, 44]. Nevertheless, the differences concerning resistance to displacement that these two thicknesses achieve have not been tested as much as their sealing ability. The push-out test is a widely accepted method to assess the bond strength of dental materials to the dentin, thanks to its reliability and reproducibility. [33, 45, 46] Some authors recommend the use of dentin sections with thicknesses not greater than 1.5 mm for push-out tests, to avoid an overestimation of the bond strength due to an increased friction area. [32, 47] However, several studies have tested POBS of hydraulic cements using thicker samples. [31, 34–36, 39] In the present study, 3 and 5 mm-thick slices were used in order to simulate the clinical situation of apical and coronal plugs, and specifically, to verify whether the thickness of the plug may influence the bond strength achieved by the different materials.

The results of the present study suggest that the displacement resistance of ProRoot MTA may not significantly change with thickness, while for TF and BD an increase in thickness results in an improvement of their resistance. These results may be applicable in the clinical setting in those situations where optimal mechanical performance is a priority, as the thickness of the plug should be taken into consideration when using these new materials.

At 3 mm thickness, no statistically significant difference was observed in the POBS values of BD and PMTA, and both materials obtained significantly higher values than TF. These findings are in agreement with those of Stefaneli Marques et al., who found no differences (at a thickness of 2 mm) between BD and PMTA. [48] Likewise, a recent study found similar mean POBS values with both BD and MTA, which were significantly higher than that of Endosequence (commercialized as Totalfill in Europe) [49], while Al-Hiyasat and Yousef observed that Biodentine had significantly higher POBS than MTA and TotalFill in samples of 3 mm of thickness. [36] On the other hand, Kadić et al. and Paulo et al. reported better results with TF than with BD. [35, 50] The contradictory results may be due to methodological differences, such as the different periods of time that the materials were allowed to set before the test was performed [35] or the different type of presentation (putty vs. paste). The authors consider that high quality clinical trials are urgently needed for a more reliable comparison of the clinical performance of different materials.

In addition to the inferential statistical analysis of the results, a Weilbull analysis was performed to compare the predictability of the tested materials. This analysis is a useful tool to study the reliability of the materials. Weibull distribution takes into consideration two main parameters: as shape and scale. The shape parameter, or Weibull modulus (m), is the slope of the line. A higher slope represents a lower variability of the feature tested. Thus, a large Weibull modulus is a highly desirable property for a dental material as it guarantees more uniform performance, and therefore a higher reliability. [40]. The Weibull scale parameter (σ0) indicates in this particular study the resistance value at which 63.2% of the tested samples fracture. Therefore.the higher this value, the more resistant to displacement is the material. [41]

TF showed the weakest POBS in plugs 3 mm and 5 mm thick and also lower predictability. This could be explained by the consistency in which the material is packaged and commercialized. The premixed consistency could cause less control over defects that may persist in the mass of the material, as suggested by Toia et al. [51]

Increasing the thickness of PMTA did not result in a significant improvement of its POBS values. However, it did affect the predictability of this material as the m parameter was higher at 3 mm (3.09) than at 5 mm (2.28). This could be explained by the higher probability of defects both in the bulk of the material, due to the high porosity of MTA [52, 53] and also by the presence of gaps in the dentin-material interface [54]. The specific need for an intrinsic water supply for optimal setting of PMTA could be responsible for the presence of defects, as increasing the thickness of the cavity makes it more difficult for moisture to reach the deeper layers. [50] Thus, the greater the thickness, the greater the probability of imperfections.

In contrast, at a thickness of 5 mm, the m parameter was higher for BD than for TF and PMTA, meaning that BD is more predictable than the other two materials. This could be caused by the low porosity and high homogeneity shown by BD [50, 52], probably due to the insoluble polymers included in its composition, which help maintain a balance between the water content and the consistency of the preparation, to obtain a more homogeneous and dense cement [11]. Also, BD has smaller particle size and shows more tags in the interface with dentin than PMTA, which may account for the higher bond strength results [31].

When analyzing the fracture pattern of all the groups, the mixed type was the most frequent in all study groups. The same fracture pattern was predominantly observed in PMTA samples in a study by Kadic et al. [50], while the most common pattern in the BD group was cohesive. Interestingly, Hiyasat and Yousef found that BD and TF were mostly associated with mixed pattern, while MTA was predominantly associated with a cohesive fracture [36]. A number of other studies were consistent at identifying cohesive and mixed as the most frequent fracture patterns [32, 33, 37, 38]. Contrary to the previously mentioned studies, other authors found the adhesive fracture pattern to be the most frequent in both MTA and BD groups [35, 45]. The differences among these results could be explained by methodological factors, such as the relative diameters of punch and cavity or the speed of the punch approaching the samples. They may also be explained, again, by the different setting time that preceded the push-out test. The adhesive type of failure is associated with low bonding strength, being the latter lower than the cohesive strength of the materials. Predominant adhesive failure suggests a poor ability of the material to adhere to the dentin, possibly due to an unfinished hydration process [35]. On the other hand, cohesive and mixed types of failure may be regarded as indicators of a higher ability to bond to the dentin wall of the studied cements.

## Conclusions

To summarize the main findings of this study, it may be concluded that TF and BD achieve higher pushout bond strength resistance when used at a thickness of 5 mm than at 3 mm, while the mean resistance of PMTA is less influenced by the thickness. At 5 mm, BD and PMTA exhibit similar resistance to displacement. However, the behavior of BD is more predictable than that of its predecessor.

## Supplementary Information

**Additional file 1**. 1. Push-out bond strength results and stadistical analysis results. 1.1 ESTADÍSTICA DESCRIPTIVA instead Descriptive stadistics. 1.2. Test de normalidad instead Normality test. 1.3. ANÁLISIS DE VARIANZA instead Bifactorial Anova Test.

**Additional file 2**. Excell Table of Raw Data.

## Data Availability

All data generated and analysed are included in this article and in the Additional files 1, 2 (data raw). Materials and data employed in this study are available and can be accessed from the corresponding author on reasonable request. Samples have been stored and are disposable. Results obtained in the Push out bond machine have been analized and the stadistical analysis has been performed employing computer program SPSS 22 (SPSS Inc, Chicago, USA) and SAS 9.4 (SAS Institute, North Caroline, USA).

## Abbreviations

BD: Biodentine
TF: Totalfill root repair paste
PMTA: ProRoot MTA White
POBS: Push out bond strength
