# Supplementary material for: Push out bond strength of hydraulic cements used at different thicknesses
Source: BMC Oral Health. 2023 Feb 7;23:81. doi: 10.1186/s12903-023-02758-w (PMC9906962; doi:10.1186/s12903-023-02758-w)
Supplement: Supplementary file 1 — Additional file 1. 1. Push-out bond strength results and stadistical analysis results. 1.1 ESTADÍSTICA DESCRIPTIVA instead Descriptive stadistics. 1.2. Test de normalidad instead Normality test. 1.3. ANÁLISIS DE VARIANZA instead Bifactorial Anova Test. [file 12903_2023_2758_MOESM1_ESM.docx]

**1. PUSH-OUT BOND STRENGTH RESULTS**

| TOTALFILL 3 MM | |  |
| --- | --- | --- |
| SAMPLE Nº | STRENGTH (N) | RESISTANCE (Mpa) |
| 1 | 23,66 | 1,794 |
| 2 | 52,4 | 3,973 |
| 3 | 40,78 | 3,092 |
| 4 | 45,92 | 3,481 |
| 5 | 37,18 | 2,819 |
| 6 | 28,26 | 2,142 |
| 7 | 16,8 | 1,273 |
| 8 | 68,08 | 5,162 |
| 9 | 32,02 | 2,427 |
| 10 | 44,8 | 3,397 |
| 11 | 44,56 | 3,378 |
| 12 | 52,54 | 3,983 |
| 13 | 105,64 | 8,01 |
| 14 | 48,42 | 3,671 |
| 15 | 70,36 | 5,335 |
| 16 | 56,88 | 4,313 |
| 17 | 83,32 | 6,317 |
| 18 | 38,64 | 2,929 |
| 19 | 71,02 | 5,385 |
| 20 | 101,62 | 7,705 |
| 21 | 82,14 | 6,228 |
| 22 | 57 | 4,322 |
| 23 | 45,18 | 3,425 |
| 24 | 17,64 | 1,337 |
| 25 | 104,72 | 7,94 |
| 26 | 52,96 | 4,015 |

Table S.1. Results of strength and resistance for samples of Totalfill Root Repair Material® paste, of 3 mm of thickness.

| TOTALFILL 5 MM | |  |
| --- | --- | --- |
| SAMPLE Nº | STRENGTH (N) | RESISTANCE (Mpa) |
| 1 | 102,82 | 4,677 |
| 2 | 34,7 | 1,578 |
| 3 | 193,16 | 8,787 |
| 4 | 43,5 | 1,979 |
| 5 | 76,5 | 3,48 |
| 6 | 98,52 | 4,482 |
| 7 | 221,24 | 10,065 |
| 8 | 77,98 | 3,547 |
| 9 | 99,78 | 4,539 |
| 10 | 173,1 | 7,875 |
| 11 | 41,1 | 1,869 |
| 12 | 97,52 | 4,436 |
| 13 | 172,28 | 7,838 |
| 14 | 72,24 | 3,286 |
| 15 | 124,88 | 5,681 |
| 16 | 151,16 | 6,877 |
| 17 | 213,98 | 9,735 |
| 18 | 86,4 | 3,93 |
| 19 | 149,02 | 6,779 |
| 20 | 150,68 | 6,855 |
| 21 | 86,48 | 3,934 |
| 22 | 144,32 | 6,565 |
| 23 | 92,76 | 4,22 |
| 24 | 144,96 | 6,595 |
| 25 | 213,54 | 9,715 |
| 26 | 45,28 | 2,06 |

Table S.2. Results of strength and resistance for samples of Totalfill Root Repair Material® paste, of 5 mm of thickness.

| BIODENTINE 3 MM | |  |
| --- | --- | --- |
| SAMPLE Nº | STRENGTH (N) | RESISTANCE (Mpa) |
| 1 | 80,22 | 6,082 |
| 2 | 116 | 8,795 |
| 3 | 78,12 | 5,923 |
| 4 | 118,58 | 8,991 |
| 5 | 92,82 | 7,038 |
| 6 | 79,9 | 6,058 |
| 7 | 48,88 | 3,706 |
| 8 | 55,2 | 4,185 |
| 9 | 141 | 10,691 |
| 10 | 119,88 | 9,09 |
| 11 | 53,92 | 4,088 |
| 12 | 60,8 | 4,61 |
| 13 | 89,78 | 6,807 |
| 14 | 67,42 | 5,112 |
| 15 | 84,7 | 6,422 |
| 16 | 84,9 | 6,437 |
| 17 | 139,66 | 10,589 |
| 18 | 63,48 | 4,813 |
| 19 | 108,96 | 8,262 |
| 20 | 98,98 | 7,505 |
| 21 | 69,12 | 5,241 |
| 22 | 43,94 | 3,331 |
| 23 | 116,36 | 8,823 |
| 24 | 131,12 | 9,942 |
| 25 | 48,36 | 3,666 |
| 26 | 88,34 | 6,698 |

Table S.3. Results of strength and resistance for samples of Biodentine™, of 3mm of thickness.

| BIODENTINE 5 MM | |  |
| --- | --- | --- |
| SAMPLE Nº | STRENGTH (N) | RESISTANCE (Mpa) |
| 1 | 110,94 | 5,047 |
| 2 | 268,06 | 12,195 |
| 3 | 100,64 | 4,578 |
| 4 | 210,1 | 9,558 |
| 5 | 165,58 | 7,533 |
| 6 | 163,84 | 7,454 |
| 7 | 131,1 | 5,964 |
| 8 | 178,14 | 8,104 |
| 9 | 230,76 | 10,498 |
| 10 | 223,16 | 10,152 |
| 11 | 201,86 | 9,183 |
| 12 | 168,82 | 7,68 |
| 13 | 214,26 | 9,747 |
| 14 | 153,56 | 6,986 |
| 15 | 149,88 | 6,818 |
| 16 | 146,92 | 6,684 |
| 17 | 192,46 | 8,756 |
| 18 | 207,94 | 9,46 |
| 19 | 198,78 | 9,043 |
| 20 | 127,52 | 5,801 |
| 21 | 100,36 | 4,565 |
| 22 | 119,8 | 5,45 |
| 23 | 187,16 | 8,515 |
| 24 | 178,08 | 8,101 |
| 25 | 259,54 | 11,808 |
| 26 | 226,94 | 10,324 |
|  |  |  |

Table S.4. Results of strength and resistance for samples of Biodentine™, of 5mm of thickness.

| MTA 3 MM |  |  |
| --- | --- | --- |
| SAMPLE Nº | STRENGTH (N) | RESISTANCE (Mpa) |
| 1 | 139,88 | 10,606 |
| 2 | 79,88 | 6,057 |
| 3 | 118,58 | 8,991 |
| 4 | 83,74 | 6,349 |
| 5 | 88,56 | 6,715 |
| 6 | 54,04 | 4,097 |
| 7 | 95,92 | 7,273 |
| 8 | 191,72 | 14,537 |
| 9 | 70,14 | 5,318 |
| 10 | 65,46 | 4,963 |
| 11 | 152,12 | 11,534 |
| 12 | 100 | 7,582 |
| 13 | 139,24 | 10,558 |
| 14 | 84,9 | 6,437 |
| 15 | 89,34 | 6,774 |
| 16 | 81,22 | 6,158 |
| 17 | 90,24 | 6,842 |
| 18 | 75,16 | 5,699 |
| 19 | 73,68 | 5,586 |
| 20 | 167,66 | 12,713 |
| 21 | 143 | 10,843 |
| 22 | 81,36 | 6,169 |
| 23 | 83,04 | 6,296 |
| 24 | 101,18 | 7,672 |
| 25 | 138,04 | 10,467 |
| 26 | 172,28 | 13,063 |

Table S.5. Results of strength and resistance for samples of ProRoot MTA® White of 3mm of thickness.

| MTA 5 MM |  |  |
| --- | --- | --- |
| SAMPLE Nº | STRENGTH (N) | RESISTANCE (Mpa) |
| 1 | 141,62 | 6,443 |
| 2 | 64,2 | 2,92 |
| 3 | 84,36 | 3,838 |
| 4 | 61,54 | 2,799 |
| 5 | 163,78 | 7,451 |
| 6 | 63,42 | 2,885 |
| 7 | 171,06 | 7,782 |
| 8 | 177,38 | 8,07 |
| 9 | 165,98 | 7,551 |
| 10 | 163,06 | 7,418 |
| 11 | 180,08 | 8,192 |
| 12 | 202,04 | 9,191 |
| 13 | 223,4 | 10,163 |
| 14 | 72,06 | 3,278 |
| 15 | 247,86 | 11,276 |
| 16 | 56,78 | 2,583 |
| 17 | 136,64 | 6,216 |
| 18 | 91,02 | 4,141 |
| 19 | 59,74 | 2,717 |
| 20 | 267,64 | 12,175 |
| 21 | 108,78 | 4,949 |
| 22 | 66,88 | 3,042 |
| 23 | 263,4 | 11,983 |
| 24 | 181 | 8,234 |
| 25 | 85,62 | 3,895 |
| 26 | 237,68 | 10,813 |

Table S.6. Results of strength and resistance for samples of ProRoot MTA® White of 5mm of thickness.

1.1 ESTADÍSTICA DESCRIPTIVA

| MATERIAL | GROSOR | N | Media | Mediana | Desv. Desviación | Desv. Error promedio |
| --- | --- | --- | --- | --- | --- | --- |
| Totalfill | 3 mm. | 26 | 4,14819 | 3,82200 | 1,902544 | ,373120 |
|  | 5 mm. | 26 | 5,43785 | 4,60800 | 2,545617 | ,499236 |
| Biodentine | 3 mm. | 26 | 6,65019 | 6,42950 | 2,203267 | ,432096 |
|  | 5 mm. | 26 | 8,07708 | 8,10250 | 2,113398 | ,414471 |
| PMTA | 3 mm. | 26 | 8,04996 | 6,80800 | 2,815383 | ,552142 |
|  | 5 mm. | 26 | 6,53865 | 6,93050 | 3,166277 | ,620958 |

Table S.7. Descriptive stadistics of the six study groups

1.2. PRUEBAS DE NORMALIDAD

The normality test was carried out and it was concluded that all the groups fulfilled the normality criteria. Next, the stadistical test for metrics were applied.

|  | | Shapiro-Wilk | | |
| --- | --- | --- | --- | --- |
| Material | Thicknesses | Stadistical | Gl | Sig. |
| Totalfill | 3 mm | ,938 | 26 | ,123 |
|  | 5 mm | ,946 | 26 | ,190 |
| Biodentine | 3 mm | ,953 | 26 | ,272 |
|  | 5 mm | ,976 | 26 | ,790 |
| PMTA | 3 mm | ,896 | 26 | ,013 |
|  | 5mm | ,912 | 26 | ,029 |

Table S.8. Normality test (Shapiro- Wilk) for six sample groups for resistance parameter.

1.3 ANÁLISIS DE VARIANZA BIFACTORIAL

A bifactorial ANOVA test is applied in order to determine if the thickness and the material have a stadistically significant impacto on the bond strength of the three hydraulic cements to root dentin. With this test determined that there is no significant interaction between the materials and the thicknesses with respect to the dependente variable resistance for TF and BD, but there is for PMTA (p=0,004).

1.4 STADISTICAL ANALYSIS FOR SAMPLES OF THE SAME THICKNESS AND DIFFERENT MATERIAL

When there was hetereogeneity of variances, robust tests of equality of means of Welch and Brown-Forsythe were carried out. The level of significance was established at p ≤ 0,05 and a confidence interval of 95%.

Stadistical analysis of the 3 mm thick groups:

In the group of 3 mm samples, stadistically significative differences were detected between the three groups of materials. To analyze these differences, a post hoc multiple comparison test of Tamhane´s T2 was performed.

Stadistically significative differences were detected between the group with the least resistance, which is TF and the other two (BD and PMTA). However there were no stadistically significative differences between the BD and PMTA (p=0,147) (table 10).

|  | Stadistics | gl1 | gl2 | Sig. |
| --- | --- | --- | --- | --- |
| Welch | 19,768 | 2 | 48,883 | ,000 |
| Brown-Forsythe | 18,583 | 2 | 67,585 | ,000 |

Table S.9. Robust tests of equality of means for groups of 3 mm samples.

| (I) MATERIAL | (J) MATERIAL | Means differences (I-J) | Deviation. Error | Sig. | Intervalo de confianza al 95% | |
| --- | --- | --- | --- | --- | --- | --- |
|  |  |  |  |  | Lower limit | Upper limit |
| Totalfill | Biodentine | -32,99615 | 7,52883 | ,000 | -51,6087 | -14,3836 |
|  | MTA | -51,45538 | 8,78814 | ,000 | -73,2700 | -29,6407 |
| Biodentine | Totalfill | 32,99615 | 7,52883 | ,000 | 14,3836 | 51,6087 |
|  | MTA | -18,45923 | 9,24617 | ,147 | -41,3463 | 4,4278 |
| PMTA | Totalfill | 51,45538 | 8,78814 | ,000 | 29,6407 | 73,2700 |
|  | Biodentine | 18,45923 | 9,24617 | ,147 | -4,4278 | 41,3463 |

Table S. 10. Post-hoc Tamhane´s T2 test for groups of 3 mm

Stadistical analysis of the 5 mm thick groups:

Robust tests of equality of equality of means between the groups of 5 mm samples were also carried out and stadistically significant differences were detected (p≤0,003) (table 11). Tamhane´s T2 post hoc multiple comparison test was performed.

Robust tests of equality of means were also carried out and stadistically significant differences were also observed between the groups of 5mm samples. The multiple comparisons test of posteriori T2 Tamhane´s was carried out (table 12). Stadistically significative differences were detected between the group with the greatest resistance, wich is BD and the least resistant which is TF (p=0,001). No stadistically significant differences were found between PMTA and TF (p=0,435), nor when comparing the first one with BD (0,130).

|  | Stadistics | gl1 | gl2 | Sig. |
| --- | --- | --- | --- | --- |
| Welch | 8,377 | 2 | 48,760 | ,001 |
| Brown-Forsythe | 6,536 | 2 | 67,685 | ,003 |

Table S.11. Robust Test of Welch and Brown-Forsythe for 5 mm samples.

| (I) MATERIAL | (J) MATERIAL | Mean differences (I-J) | Desv. Error | Sig. | Confidence interval 95% | |
| --- | --- | --- | --- | --- | --- | --- |
|  |  |  |  |  | Lower limit | Upper limit |
| Totalfill | Biodentine | -58,01154 | 14,26186 | ,001 | -93,2847 | -22,7384 |
|  | MTA | -24,19692 | 17,51306 | ,435 | -67,5296 | 19,1357 |
| Biodentine | Totalfill | 58,01154 | 14,26186 | ,001 | 22,7384 | 93,2847 |
|  | MTA | 33,81462 | 16,41017 | ,130 | -6,9314 | 74,5607 |
| PMTA | Totalfill | 24,19692 | 17,51306 | ,435 | -19,1357 | 67,5296 |
|  | Biodentine | -33,81462 | 16,41017 | ,130 | -74,5607 | 6,9314 |

Table S. 12. Post-hoc T2 Tamhane´s Test for 5 mm groups.

1.5 STADISTICAL TEST FOR SAMPLES OF THE SAME MATERIAL AND DIFFERENT THICKNESSES

T Student´s Test was performed to analize differences between strength and resistance between samples of 3 and 5 mm for each material (table 13).

| MATERIAL | | | Levene Test of iguality of variances | | T test for medis equality | | | | | | |
| --- | --- | --- | --- | --- | --- | --- | --- | --- | --- | --- | --- |
|  |  |  | F | Sig. | t | gl | Sig. (bilateral) | Medias difference | Estándar error difference | 95% confidence interval of the difference | |
|  |  |  |  |  |  |  |  |  |  | lower | upper |
| Totalfill | STRENGTH | Equal variances are assumed | 20,119 | ,000 | -5,390 | 50 | ,000 | -64,82154 | 12,02575 | -88,97596 | -40,66712 |
|  |  | Equal variances are not assumed |  |  | -5,390 | 34,663 | ,000 | -64,82154 | 12,02575 | -89,24358 | -40,39950 |
|  | RESISTANCE | Equal variances are assumed | 4,097 | ,048 | -2,069 | 50 | ,044 | -1,289654 | ,623262 | -2,541512 | -,037796 |
|  |  | Equal variances are not assumed |  |  | -2,069 | 46,287 | ,044 | -1,289654 | ,623262 | -2,544005 | -,035303 |
| Biodentine | STRENGTH | Equal variances are assuumed | 5,532 | ,023 | -8,360 | 50 | ,000 | -89,83692 | 10,74548 | -111,41985 | -68,25399 |
|  |  | Equal variances are not assumed |  |  | -8,360 | 41,966 | ,000 | -89,83692 | 10,74548 | -111,52270 | -68,15114 |
|  | RESISTANCE | Equal variances are assumed | ,061 | ,806 | -2,383 | 50 | ,021 | -1,426885 | ,598743 | -2,629496 | -,224273 |
|  |  | Equal variances are not assumed |  |  | -2,383 | 49,914 | ,021 | -1,426885 | ,598743 | -2,629548 | -,224221 |
| MTA | STRENGTH | Equal variances are assumed | 13,966 | ,000 | -2,428 | 50 | ,019 | -37,56308 | 15,46998 | -68,63544 | -6,49071 |
|  |  | Equal variances are not assumed |  |  | -2,428 | 38,164 | ,020 | -37,56308 | 15,46998 | -68,87600 | -6,25015 |
|  | RESISTANCE | Equal variances are assumed | ,684 | ,412 | 1,819 | 50 | ,075 | 1,511308 | ,830933 | -,157670 | 3,180286 |
|  |  | Equal variances are not assumed |  |  | 1,819 | 49,326 | ,075 | 1,511308 | ,830933 | -,158236 | 3,180851 |

Table S.13. T Test for the six groups for strength and resistance.

1.6 RELIABILITY ANALYSIS: WEIBULL TEST

The Weibul distribution depends on the form a (m) and scale parameters (σ_0_).

|  | m = form Weibull | | | | σ_0_ = scale Weibull | | | |
| --- | --- | --- | --- | --- | --- | --- | --- | --- |
|  | Value | Standard value | Confidence interval 95% | | Value | Standard value | Confidence interval 95% | |
| Totalfill 3mm | 2.3832 | 0.3584 | 1.7748 | 3.2001 | 4.6928 | 0.4083 | 3.9571 | 5.5652 |
| Totalfill 5mm | 2.3531 | 0.3674 | 1.7328 | 3.1955 | 6.1540 | 0.5408 | 5.1802 | 7.3108 |
| Biodentine 3mm | 3.3940 | 0.5196 | 2.5143 | 4.5817 | 7.4208 | 0.4534 | 6.5832 | 8.3650 |
| Biodentine 5mm | 4.3754 | 0.6692 | 3.2421 | 5.9050 | 8.8744 | 0.4199 | 8.0884 | 9.7368 |
| PMTA 3mm | 3.0967 | 0.4536 | 2.3240 | 4.1265 | 9.0158 | 0.6064 | 7.9022 | 10.2863 |
| PMTA 5 mm. | The2.2864 | 0.3593 | 1.6803 | 3.1111 | 7.4156 | 0.6716 | 6.2095 | 8.8560 |

Table S. 14.Table describing parameters form and scale of Weibull´s test for the six study groups and their confidence intervals 95%.

Weibull analysis for hydraulic materials of 3 mm:
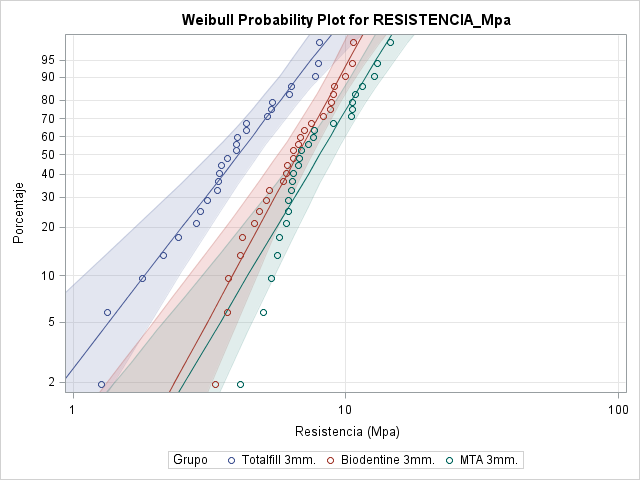


Figure S.1. Weibull probability plot for resistance analysis for the 3 mm groups

Weibull analysis for hydraulic materials of 5 mm:


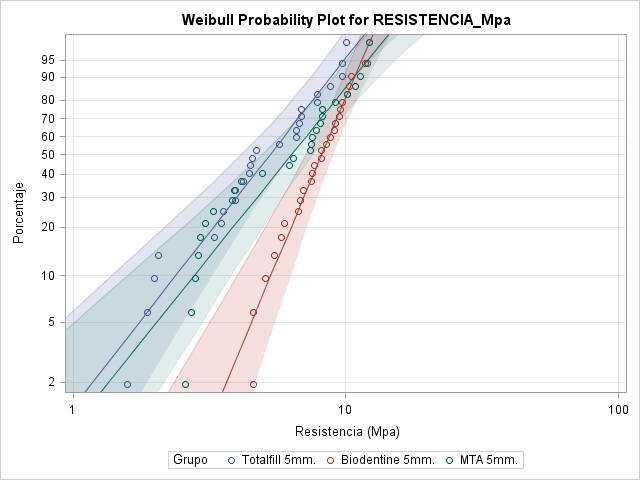


Figure S.2.Weibull probability plot for resistance analysis for the 5 mm groups
